# Sii-Mobility: An IoT/IoE Architecture to Enhance Smart City Mobility and Transportation Services

**DOI:** 10.3390/s19010001

**Published:** 2018-12-20

**Authors:** Claudio Badii, Pierfrancesco Bellini, Angelo Difino, Paolo Nesi

**Affiliations:** Department of Information Engineering, DISIT Lab, University of Florence, 3, 50139 Florence, Italy; claudio.badii@unifi.it (C.B.); pierfrancesco.bellini@unifi.it (P.B.); angelo.difino@unifi.it (A.D.)

**Keywords:** smart city, mobility, IoT applications, safety critical

## Abstract

The new Internet of Things/Everything (IoT/IoE) paradigm and architecture allows one to rethink the way Smart City infrastructures are designed and managed, but on the other hand, a number of problems have to be solved. In terms of mobility the cities that embrace the sensoring era can take advantage of this disruptive technology to improve the quality of life of their citizens, also thanks to the rationalization in the use of their resources. In Sii-Mobility, a national smart city project on mobility and transportation, a flexible platform has been designed and here, in this paper, is presented. It permits one to set up heterogeneous and complex scenarios that integrate sensors/actuators as IoT/IoE in an overall Big Data, Machine Learning and Data Analytics scenario. A detailed and complex case-study has been presented to validate the solution in the context of a system that dynamically reverse the traveling direction of a road segment, with all the safety conditions in place. This case study composes several building blocks of the IoT platform, which demonstrate that a flexible and dynamic set-up is possible, supporting security, safety, local, cloud and mixed solutions.

## 1. Introduction

Since the rapid spread of urbanization across the Western world in the 1950s, mobility has driven important aspects for growth and progress. At the beginning mobility gave people the freedom to move around with new means of transportation, independently from where they live or they work, while today due the strong linkage of traffic and communication systems and the fact that the infrastructure is stressed to its limits, it is essential to think about alternative, dynamic routes and paths and more efficient/optimized transportation systems. Smart traffic planning, innovative public transport systems and personal multi-modal routing (for the citizens of city that have already embraced this new paradigm) are central for a complete interconnected infrastructure. Less traffic jams and near-to-zero emissions with smart means of transportation have been demonstrated to produce relevant impacts on the environment and quality of life in smart cities, increasing positive virtuous habits among the population. Nowadays, the development of key enabling technologies, such as Internet of Things (IoT) and Internet of Everything (IoE), is driving even more rapidly the growth of sustainable ecosystems. The switch to the IoT/IoE paradigm is going to dramatically change (again) the way the citizens and city operators interact with its nearby infrastructure: how they move, how they get energy, how they make decisions, and how the city entities are managed and controlled [1].

The pervasiveness of IoT Devices in the physical world defines the stringent connectedness between the real and virtual worlds. An IoT Device can be seen as a hardware device that integrates some kind of sensors/actuators eventually embedded in a real-world object to enhance its capabilities or that can stay alone to sensing/inform directly the environment in which it is located. The IoT devices are usually (inter)connected to/with the main Internet or via a local area network, depending the scope of the smartness to implement (global or local). The IoT can be described as *“a dynamic global network infrastructure with self-configuring capabilities based on standard and interoperable communication protocols where physical and virtual ‘Things’ have identities, physical attributes, and virtual personalities and use intelligent interfaces, and are seamlessly integrated into the information network”* [2] or *“a global infrastructure for the information society enabling advanced services by interconnecting (physical and virtual) things based on, existing and evolving, interoperable information and communication technology”* [3].

IoT architectures can be described as independent IoT ecosystems that can be physical, virtual or a hybrid mix of the two. They consist of a list of active physical devices, sensors, actuators, services, communication protocols and layers, final users, developers and interface layers. Several functional blocks are defined in an IoT system, even if a commonly agreed conceptualization is not found, but several different approaches are usual considered: a three-layer architecture constituted by Application, Network and Perception layers [4]; a five-layer architecture including also Business and Process layers [5]; cloud and fog systems [6]; and social IoT paradigms [7]. The development of next generation IoT infrastructures is still at its early stage and relevant progress is expected to be made in the next years. According to Gartner [8], by 2020 up to 20.4 billion IoT devices will be connected together. New tools to support the new paradigm are needed: smart management of the resources, better security for population, healthcare and engagement of the citizens in their everyday activities are some examples of the different scenarios that can be unlocked with an embedded IoT ecosystem support [9].

This paper presents the work performed on defining a smart city architecture to enable the integration and implementation of a set of different and heterogeneous scenario for the IoT/IoE paradigm in the context of mobility and transport, where the accent on safety critical aspects is relevant. Thus, a set of requirements and use cases are discussed and addressed, and thus, a complete experiment is detailed: a smart modality to manage a dynamic management of direction of a one-way road segment, including dynamic signage and corresponding synchronizations. This example employs the use of several modules of the architecture and puts in evidence how a local solution can be implemented beside a complete cloud architecture, also respecting the safety critical constraints. The problem and the solution are explained to catch the value of having a complete platform from the sensing/actuating activities towards a programmable environment where the city operators can implement their strategies [10], in a fully distributed manner.

## 2. Requirements and Architecture

Most of the smart city solutions target several different aspects of the infrastructure to improve the sustainability of their services [11]. Several use case scenarios have been taken into consideration and some of them belong to very different applications’ domains, like healthcare management, smart mobility, citizen’s security, efficient farming, etc., [12,13]. In order to enable the city operator and to introduce smart solutions capable of helping citizens in their daily activities, a set of heterogeneous and integrated tools are needed. These solutions have to be capable to support different scenarios in a smart and integrated way, to provide a mechanism to senses the context of the city, to drive the data in a singular or aggregated way, to provide at data scientists a set of tools for data analysis and a programming environment to computer scientist. A smart city platform should be capable to inform both city users (citizens, students, commuters, workers, etc.) and city operators, to collect their feedback about city infrastructures and services, to provide an integrated info-portal for ad-hoc and personal visual applications (City Dashboards) and finally to provide to the platform’s admins a way to monitor the functionalities of the solution.

The most mature IoT architecture that is available for trial and that represent the background of our research is the Amazon Web Services (AWS) IoT [14], a complete cloud solution for IoT provided by Amazon: this platform deploys their service to be simple and secure and fully integrated with other tools of the Amazon ecosystem. The IoT architecture is represented by a cloud solution that on the edge side (Device Gateway) provides a system of shadowing to cope with intermittent connectivity of the IoT devices. An internal Rule Engine allows developers to write simple algorithms to process the data carried out from the sensors via several protocols (i.e., MQTT over TLS). A Registry Unit is implied to collect any information of the IoT Device and track their functionalities. On the contrary, a different approach has been taken from Microsoft solution called Azure IoT Suite [15], where just two layers are present: the IoT solution backend represents a set of Azure services (machine learning and analytics) and the presentation layer involves the visual presentation of the data. Microsoft architecture enables the communication of the IoT device to the Azure cloud via a predefined cloud gateway or via an IoT hub that includes an identity registry for managing the identity and authenticating the devices. Several other IoT solutions are available, but none of them have reached maturity compared to the two previously mentioned ones: the solution released by Google called BRillo/Weave, the IoT system for ARM microcontroller called ARM mbed IoT, the IoT platform promoted by Ericsson (Calvin), by Apple (HomeKit) and by IBM (Watson IoT). Important to notice, the Blockchain [16] paradigm is raising more and more interest to support the IoT architecture idea, providing decentralization of data and supporting dynamic and complete distributed solutions. Several approaches have been proposed [17], even if problematic aspects still need to be resolved [18]. In the context of mobility and transport, with a wider view of the mobility and transportation aspects of the city, Intelligent Transport Systems (ITS) should be capable of managing traffic lights, dynamic signage, speed control systems, smart parking, car sharing, etc. The IoT revolution may also impact mobility and transport applications, in a distributed manner. An IoT platform for a smart city must support scenarios that focus on the way the citizens interact with their infrastructure (in terms of streets, roads, cycle paths, public transport stations, parking, etc.) [19], the way the city operator of the mobility and transport unit of the city programs, configures the transportation infrastructure and how it is managed/monitored daily. 

The main requirements identified in the above described context are reported as follows. So that, an integrated solution for IOT/IOE has to be capable to:

- collect a big amount and heterogeneous data from the city infrastructure [20] in a continues way and support different ways for data injection (push, pull, on-demand, offline); support local scenario (not connected to the Internet) and completely on-cloud architecture;
- assure an architecture that is complete and consistent due to the fact that some scenarios can be safety-critical, and thus the communications among IoT elements, and with the control room (traffic congestion area, event mass moving, pedestrian cross paths) have to be secure; as well as their implementation has to be capable to cope with eventual lacks of communication/ connection; in same cases real-time or near-real-time safety mechanisms have to be provided [21];
- guarantee that the solution is distributed and scalable with respect to the points of controls, and that they may be capable to continue to work even if the connections with the control room, or each other are lost; the control room may be used to force situation and monitoring in the presence of connection, but all components have to work even without this connection;
- guarantee that the data are managed properly, in respect of their ownership and the explicit signed consents they gave; protect against external intrusion and data tampering, man-in-the-middle attacks and data sniffing; monitor suspicious activities and enable inspection from law enforcement agencies; inform in case of a breach [22];
- store these data in a distributed storage system and index them on the basis of different constraints imposed by the domain application [23]; support an efficient way for data retrieval and provide a complete system of backup and solution for disaster recovery;
- provide a programmable environment to define algorithms for machine learning and data analysis, able to extract characteristics from the collected data (aggregation of data) [24] and elaborated them in order to implement predictive capabilities for guessing their future evolution: smart parking, predictions on traffic flow, traffic flow reconstruction;
- enable visual presentations of data via a set of dashboards composed by widget components that a city operator can manage graphically; these widgets have to be manageable/configurable by a person that may not know deeply the technicisms behind the IoT infrastructure.

In the context of mobility and transport, the data exchanged and managed by IoT devices, toward the infrastructure in which the local computing capabilities, can be used via an IoT Edge (an IoT device with enhanced capabilities of local computing), so that the features of those devices should locally provide have been identified, and are reported as follows. They have to cope with:

- dynamic signals on the road with respect to the conditions of the local sensors; display information to drivers and to people on the street: speed limits, presence of active/inactive smart speed meters, digital road display about the traffic condition and best solution for public transportation;
- collect information about traffic flow sensors on the city road (how many cars passed a specific segment during the last time slot, their mean speed, density of big trucks, cars, motorbikes, etc.), estimate the traffic status in terms of density of equivalent vehicles, thus abstracting from vehicle kind;
- people flow moving in crucial areas based on cameras, lasers, Wi-Fi, Bluetooth by sensors counting people density. For example, placed in front of a bus/train station, in front of an important museum; counting the people flow in some strategic areas of the city, in front of gates or around busy common walk paths [25];
- implement traffic direction changes on the road with respect to the local conditions or on demand.

Other activities may need to be performed/delegated remotely on the cloud in a mobility and transport central station and control room such as:

- predictions of: free car parking slots, delay of busses at bus stops, bikes to be shared from a given point, traffic flow at some points;
- display information to control room operators to alarm in the case of early warning conditions or for current operations;
- traffic flow: capture and visualize the current global traffic situation, predict congestion situations, suggest alternative ways to reach a point of interest [26];
- video analysis: suggest alternative paths for tourists to avoid congestion areas (long queues) and to promote new and alternative tours;
- public transport: describe in a visual and integrated way the complete public transport infrastructure, enable queries on maps (when a bus will arrive at destination, how long it may take to commute to work) and support multi-modal routing (from here to there using different kinds of public transportation); integrate information of traffic flow to provide better predictions;
- parking: track the availability of city parking areas, their weekly/daily use, suggest free parking places in the areas a citizen usually visits [27]; predict a parking place in a future assessment or parking areas without any restriction due to street cleaning, public or private events;
- user behavior and engagement: analysis of citizen behavior in the use of the city infrastructure, highlight unsustainabke behavior and propose habit changes to promote virtuous ones [28]; track if the suggestions are followed and rewards the “good” citizens;
- actuator: manage smart traffic boards for dynamic setups or to inform citizens about congestion when disruptive events occur.

**Figure 1:**
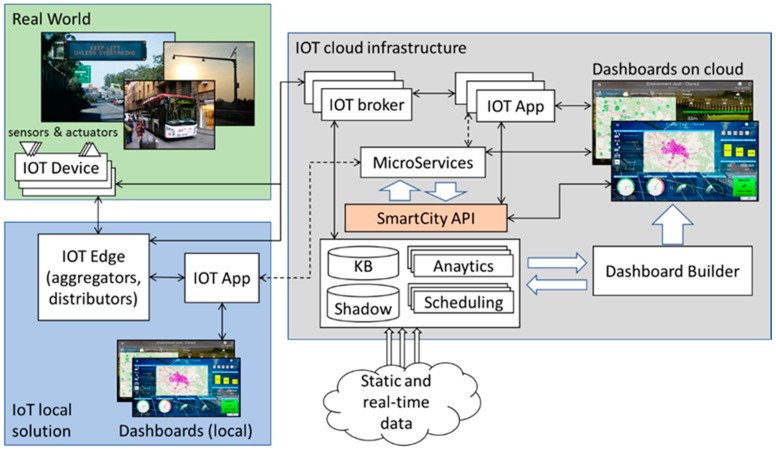
High level architecture of our IoT solution.

According to the above identified requirements and rationales the activity on Sii-Mobility project and challenge started. Then the designed and implemented architecture resulted to be flexible enough to support the above requirements and use case scenarios, on IoT edge scenarios (local IoT solutions, independent from the Internet connection) and cloud (enriched with control room support). In the context of this paper, we focus our attention on IoT Edge cases with some integration to cloud computing and control rooms. Figure 1 highlights the architecture on the basis of its major macroblocks. Starting from the left upper part, the Real World and the environment are inter-connected to our infrastructure via a set of IoT devices that are enriched onboard with a set of different sensors and actuators. Thus, a multitude of different IoT devices are supported by the proposed ecosystem: they can be classified on the basis of their energy consumption and distance in terms of network hops to our main backbone. The IoT devices can eventually be integrated in real world objects to provide some sort of smartness and connectivity. An IoT device can be implemented by using a simple microchip (i.e., an esp8266 with microcontroller capabilities) that communicates a set of different data values via messages with some protocol and format (in our cases mainly NGSI, MQTT). It can be enriched, for example, with a simple sensor (a button) that can be connected to our backbone or to an external aggregator device (IoT Edge) via a wireless network interface towards an Internet access point with standard communication capabilities (IEEE 802.x), or with patented radio communication technologies (such as LoRa, SigFox, OneM2M, …). Most of this kind of IoT devices have no or low capabilities of embedding logic and are just used to collect and transmit data from their embedded sensors to the platform or to perform some action to their embedded actuators on the basis of the data received. On similar capabilities, a microcontroller board (i.e., Arduino) can be used: it adds a slightly bit more dynamic setup, with aggregation capabilities between different type of sensors and actuators that can be integrated. These IoT devices are low energy consumption and usually works using strategies for sending data when they are significant or on-demand. 

The data generated from the sensor or received by an actuator can be sent/received (via its supporting IoT device) to/from an IoT Edge that acts like an aggregator/distributor/gateway and that introduces the possibility to define some kind of logic (IoT application) to enable IoT local solutions. IoT Edge devices provide more computational capability and can be seen in substance as small single board computers (i.e., Raspberry PI). They can be directly connected to a wireless network via a more powerful link, using a little more energy, and can be installed where remote powering is available. They can act as sensors/actuators, as well as aggregators among of the lower level former IoT devices or as router/gateway towards cloud IoT smart city backbone. Handheld personal computers (i.e., smartphones, tablets) can be regarded as IoT Edge devices and are also supported: they are similar devices to the previous class, but with an embedded network connection to the Internet via 3G-5G capabilities, so they can act and communicate directly to the smart city infrastructure, even if the energy consumption has to be taken more in consideration; more generally any personal computer and Internet device can be integrated in the smart city IoT/IoE solution, whenever energy consumption and price is not an issue. Finally, virtual sensors and actuators can be also created directly in the cloud infrastructure for offering operators and users a way to send messages and show results on some user interface, so that, a button on the user interface of an IoT application can be regarded as virtual sensors generating a message to an IoT broker. In IoT Edge raw data (or the elaborated ones) can be represented locally (in terms of closeness to the sensors/actuators) via local dashboards. 

In some cases, the logic (IoT application) can use external services formalized as microservices and exposed by the smart city platform. They are remotely exploited and, of course, when the Internet connectivity is lost, the application has to adapt the logic to work with the internal data and services according to its purpose. The microservices provide by smart city infrastructure are implemented using the SmartCity API [29] that works directly on top of Km4City Knowledge Base and a set of additional tools (for sake of readability, here we just presented two main macro-blocks: analytics and scheduling; for a more detailed description refer [30]).

In case an on-cloud solution it is chosen, the data can flow toward/from smart city IoT cloud infrastructure by means of:

- a set of Context Brokers (Mosquito MQTT, IOT Orion Broker NSGI, RabbitMQ, etc.) which collect the data and allow other IoT applications to make subscription to services;
- IoT brokers implement a shadowing and indexing system to enable the collection of all changes performed on IoT devices sensors/actuators and enable those data to be accessed by means of Smart City API for the IoT applications, data analytics and dashboards; this means that when the IoT devices go off-line the system is capable to provide the last value and also the historical values that can also be used for publishing then as new data sets on Open Data Portals, automatically;
- IoT Edge devices on the field (IoT local solutions) which may have embedded IoT applications also working with microservices and local dashboards;
- IoT applications on the cloud, which may subscribe to IoT broker data, exploit microservices and thus services about IoT data shadowing and indexing, data analytics, scheduling or processes, and dashboards.

Several other management tools are available to the IoT application programming logic in the backbone of the solution. In the context of this paper, the Dashboard Builder interfaces the IoT application to the visual presentation of the data (dashboards on the cloud). This module enables the city operator to compose in a graphic dynamic and intuitive manner the visual presentations of data using a set of widgets that can be defined on top of raw or structured data. The collection of graphic widgets can be easily extended by developing specific PhP modules. The dashboard builder as the whole infrastructure and tools are Open Source [31] on GitHub.

The way the sensors/actuators are connected to the overall structure [32] via their IoT dDevice, which IoT infrastructure’s services are used by the IoT application programming logics, and what is the best topology to connect the sensing to the elaboration side, strongly depends on the target scenario to implement. And, as well as, on the energy availability around the sensors, on the presence of an Internet connectivity (and its relative QoS) and on the required security (sensibility of the transmitted data). 

The proposed IoT architecture of Sii-Mobility on cloud is the backbone of the solution. Completely virtualized on the Internet, it is the boilerplate where a city operator interacts via a set of graphical tools to program algorithms for data aggregation, data analytics, and thus realizing solutions for computing predictions, anomaly detection, origin destination matrices, trajectories, etc. The data produced by this calculation can be also injected back in the solution knowledge base for further analysis or and exposed to the personal dashboards for graphical presentation.

Important to note, the data generated from the IoT devices are linked to the information about the user that own the device, so the platform is able, whenever in any tools of the ecosystem, to enforce and assure that a data is threaded properly and presented just when/where the owner of the data gave his explicit consent. The data from a sensor are initially set private to the owner and thus he/she is the only one that can visualize and utilize the data. Techniques of identification and authorization are implemented in any of the tools of the proposed solution exploiting existent service when it is possible (i.e., to provide a user single point of authentication we employ the use of the open-id connect protocol) or implementing new ad-hoc solutions where there was no availability (i.e., to verify the device authorization to access an Orion broker—for example for a subscription—via a programmable firewall compliant with NGSI protocol). In the communication all data are encrypted to assure no other people can access the sensitive information, and the connection protocol is also signed by a certification authority to prevent tempering (identity stealing). Thus, any transmission between the tool’s component is protected using secure channels. Rule management for different kinds of rights and organization in groups for the classification of the users permit a flexible architecture to better organize the permissions in the overall managements of the user privacy. Finally, a delegation system is also available to enable the users to expose their data (providing specific right consent). The right management is enforced at level of IoT devices and single IoT data (delegated device/datavalue), and can be delegated to other users, or to anyone (anonymously on public).

## 3. Materials

### 3.1. IoT Devices

In this research two Raspberries Pi and an Android tablet were used. In these three devices we installed Node-RED software because through this program is possible to create applications for IoT in a flow-based programming mode. The Raspberry Pi are two “Raspberry Pi 1 Model B+ V1.2” with Raspbian operating system (based on the linux distribution Debian Stretch 9.3 and with kernel 4.9.59+), Node-RED v.0.18.4 and NodeJs v.8.10.0. Various sensors can be connected to the Raspberry Pi, for example DHT11 or DHT22 sensors that can measure temperature and humidity values from the surrounding environment and send data to our IoT broker platform (Fiware Orion Broker) via Node-RED.

**Figure 2:**
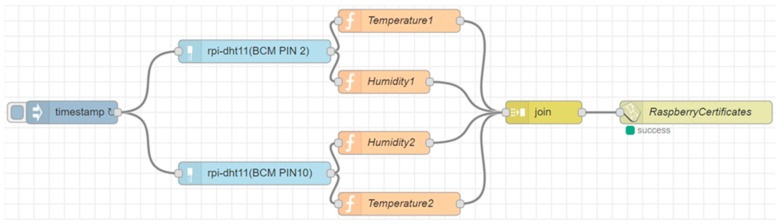
Example flow that sends data from two DHT11 sensors (humidity and temperature) to a Fiware Orion Broker.

As it can be seen in Figure 2, the flow that reads the values of the sensors (two DHT 11 connected to the GPIO of a Raspberry PI board) is very simple and intuitive. The last block allows to send the data, compliant to the Next Generation Service Interfaces (NGSI) protocol, in a protected way over HTTPS to our IoT broker that works as an endpoint for secure data entry. In addition, it is possible to insert certificates that identify the IoT device that is sending the data to create a mutual authentication flow between the IoT device and the IoT broker.

The flow makes a call to the IoT broker like this:

curl -H “Content-Type: application/json” -X POST -d “{ “contextElements”: 

[ { “type”: “EdgeDevice”, “isPattern”: “false”, “id”: “RaspberryCertificates”, 

\“attributes\”: [{“name”:“temperature2𠇌,“value”:“27.00”}, {“name”:“humidity2”,“value”:“46.00”}, {“name”:“temperature1”,“value”:“25.00”}, {“name”:“humidity1”,“value”:“54.00”}], “updateAction”: “APPEND” }” https://broker1.km4city.org:8080/v1/updateContext?elementid=RaspberryCertificates

It is possible to see that the data (compliant to NGSI protocol) are sent to the IoT broker within a JSON that contains the identifier of the entity bound to the device, the type of the entity and the data coming from the sensors that must go to update the status (or the context) of the entity saved on the Orion Broker.

**Figure 3:**
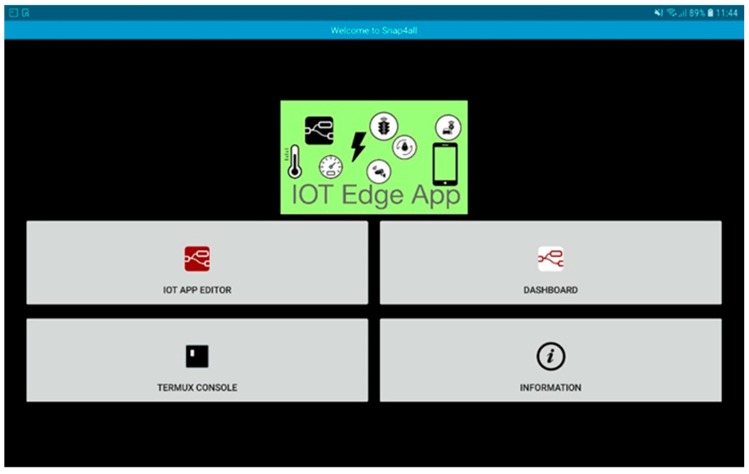
Screenshot of the application that allows to turn an Android tablet into an IoT Device.

The tablet is a Samsung Galaxy Tab S4 with the Android operating system version 8.1.0 Oreo in which a modified version of the Termux application (see https://termux.com) has been installed that allows one to install and start Node-RED directly at the boot of the device (see Figure 3).

**Figure 4:**
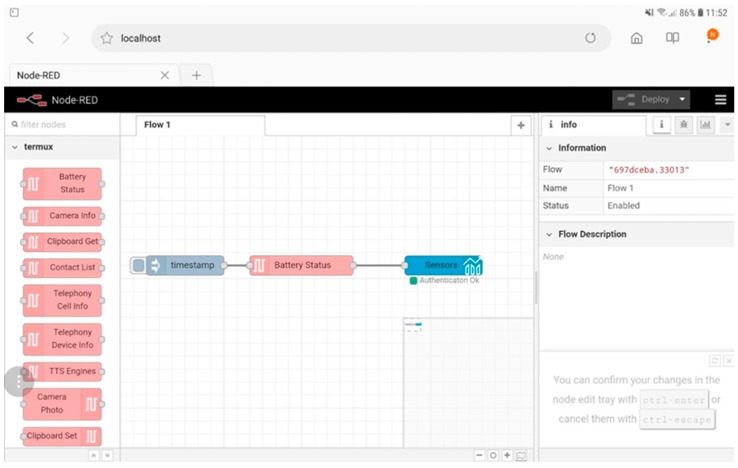
A flow that runs within the tablet and allows reading data from available sensors to send it to a private dashboard on the cloud. In the case of the Android tablet, it is possible to retrieve information through the sensors inside the phone, such as accelerometer, GPS antenna, gyroscope, WiFi antenna, battery sensor, etc.

For example, in Figure 4 it is possible to see how an application can read the battery status of the device and send the data on a dashboard hosted on cloud, protected with his personal credentials and displaying a time trend graph (note that the flow is running locally within the tablet).

### 3.2. IoT Applications

The IoT applications represent the flows that can be created through Node-RED. This tool installed by default within the Raspbian operating system (can also be installed on Windows or any other Linux distribution, like termux on Android) allows to easily create applications that permit to interact with the IoT device and make them communicate with each other automatically in an event-driven mode. 

IoT Applications can be run directly within IoT devices (Raspberry Pi, Arduino, Android devices), for example to send data to other IoT applications or IoT Orion Brokers, as shown in Figure 2 and Figure 4. They can run within the IoT cloud infrastructure to have more resources available (in terms of computing capacity and memory) and run applications involving multiple values from sensors collected from different IoT devices simultaneously. 

The nodes that are initially located within the flow development environment are useful to create for example REST API and simple local dashboards to perform actions through buttons or to view data on a graph, but it is possible to download additional nodes developed by the community that revolves around the Node-RED project.

The architecture described in this research increases the potential of the IoT applications that can be created through Node-RED software. As seen in the examples in Figure 2 and Figure 4, it is possible to use the microservices offered by our platform to add security and functionality to the IoT applications. In the case shown in Figure 2, it is possible to send data only if the IoT device has the certificates and or credential’s keys needed to be authenticated and authorized by our firewall otherwise no data can be sent. This ensures that the data received from the IoT broker has been sent just by the authorized devices. In the case shown in Figure 4, the IoT device can send the data directly to a dashboard located in the cloud and therefore it is possible to make it usable wherever the city operator can be found and not only locally as is the case with the default dashboards provided by Node-RED (they can also be accessed from outside a local network but the work to be done is not in the possibilities of the average user). As in Figure 2, also in the flow of Figure 4 the city operator must authenticate himself inside the node configuration otherwise he cannot create dashboards and cannot send data on the platform.

### 3.3. Dashboards

Once the data from the sensors has been retrieved, it may be useful for the city operator to have tools available to create dashboards that show this data in a simple and intuitive way. The architecture described in the previous chapter provides the dashboards that can be used to show raw data coming directly from the IoT devices that send it to the IoT broker or to show processed data within IoT applications: usually the processed data comes from IoT applications that are in the cloud because the IoT devices do not have enough resources to perform complex calculations on the data.

To allow data to be sent securely from the IoT devices to the dashboards in the cloud, special nodes have been developed (like the “time-trend” node on Figure 4) which, through authentication with username and password, allow the choice of an existing dashboard on which to create the new object or the creation from scratch of a new dashboard to be dedicated to the data you want to send. If the city operator cannot be authenticated, it cannot send data to the dashboards on cloud.

To allow more flexibility in the creation of the dashboards even for users who are not familiar with the IoT applications, it is possible to create them by choosing which data to show using a dashboard builder that will retrieve the data directly from the IoT brokers.

In addition, in order to not only create passive dashboards, nodes have been developed that allow to have actuators within the dashboards whose signals are sent to certain IoT applications to perform previously developed actions, when the buttons on the dashboard are pressed.

Note that while the data coming from and going to the IoT devices pass through the IoT brokers with NGSI protocol, the actions that are sent through the actuators in a dashboard to the IoT applications use a communication made with a Secure Web Socket Server to exchange information.

## 4. Experiment Key Study

**Figure 5:**
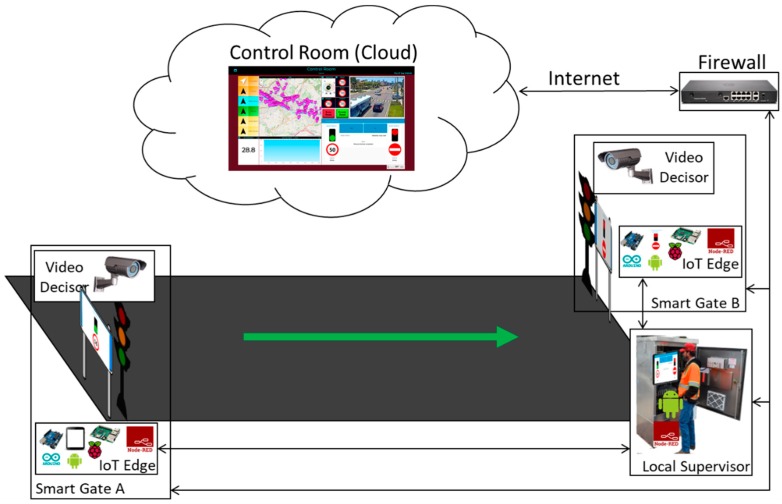
Configuration of a one-way variable system on a road segment.

In this experiment, a one-way variable road segment is taken into consideration: a road segment in which the direction of traffic flow can be dynamically inverted at any time according to the needs of the city, under the decision of the city operator managing the section. As depicted in Figure 5, the solution consists of a number of elements to manage the traffic and their management by means of a sect of interconnections. The main requirement, of course, is to be able to reverse the direction of travel without causing any accidents, putting in critical condition the drivers on the road.

There are no stringent real-time requirements, but the system is expected to respond within a reasonable time to the signals and actions that must be taken by the various subsystems. If the city operator requires an action and it is not satisfied in the time of about 5 s, a message can be displayed that something has gone wrong to warn the operator and allow him to intervene to solve the problem. The reason for not making the real-time requirement too stringent is due to the system that is developing: if the first signal does not reach its destination, the Smart Gates remain in their initial configuration (one with “Access Allowed” signal and the other with "Prohibited Access" signal, if the subsequent signals do not reach their destination there are no problems because the Smart Gates have been put in “Prohibited Access” mode and remain so until a manual intervention.

**Figure 6:**
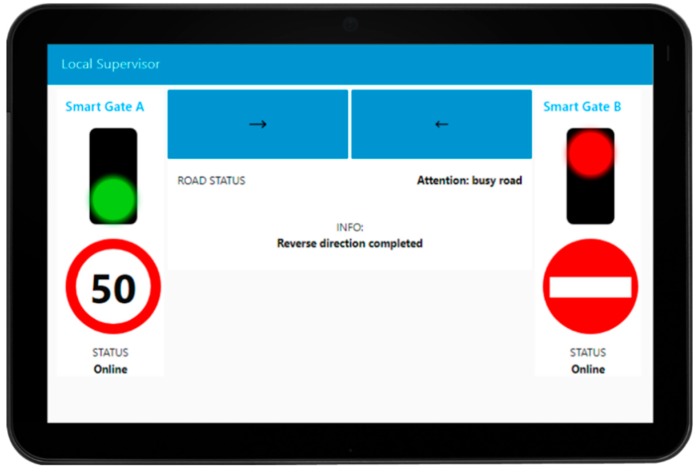
The interface (Dashboard) used to reverse the direction of travel on the road segment.

The system developed consists of two smart gates located on both sides of the segment to be managed, realized with a traffic light flanked by a display where information is shown to the drivers. The smart gates are controlled and connected to an IoT Edge each (they could be Android device as well as Raspberry Pi or Arduino) that contain the IoT app logic to control the red-light, and the messages showed on the dynamic plate display (informing that the road cannot be accessed, or providing speed limit, etc.). The two IoT Edge devices, in turn, are connected to a Local Supervisor IoT Edge device (also an Android Device) in the box of the smart gate system (usually on the side of the road segment) that can be used by the authorities to reverse the travel’s direction of the segment under consideration. This local supervisor shows a simple and intuitive visual interface via a dashboard (see Figure 6) and allows the reversal of the road with a simple press of a button in the interface.

The information coming from the smart gates through the Raspberry Pi and the commands sent from the local supervisor must have a high reliability and the city operator must be sure that what he/she is seeing in the local supervisor is what the smart gates are showing to drivers at that precise moment. For this reason, the connection between the elements of the system are preferably performed by wired to guarantee higher reliability, but connections 5G would be a good replacement for the future. In the experiment, as discussed in the Materials section, were used “Raspberry Pi Model B + V1.2” that have an Ethernet port on the card and then the connection was made with an Ethernet cable that goes from the smart gates inside the box where the Android tablet is located. Being inside a traffic light system the power supply is directly supplied by the cables that arrive at the system from the public network.

Depending on the geometry of the road segment, one or more cameras have to be installed, to be used by the Video Decisor subsystem to decide whether at a given time it is possible or not to complete the reversal operation; by controlling if the road is free of vehicles or whether the road is busy. In that case, the system has to wait until it become empty.

The smart gates always have a complementary aspect: free access on one side and no access on the other. When switching, it is needed to check that the road is empty: the safety of people is at stake, so the software cannot be decided itself on it, but needs active human help.

Clearly, the decision to reverse the one-way direction of a road can only be implemented safely if there are no vehicles in transit at that time. For this reason, a Video Decisor is used to detect the state of “Empty Road”. This module is not directly connected to the integrated actuator but to the control room for safety reasons. In fact, it is not possible to rely on the judgement of an intelligent electronic device to acquire information that, if incorrect, has a direct impact on the safety of people. The information must necessarily be confirmed by a human operator on the control room.

For these reasons, the Video Decisor communicates directly to the control room, transmitting visual information based on which the operator can confirm and authorize the switching. The overall operational sequence of actions consists of:

- setting up of both actuators at the ends of the road in a state of "Prohibited Access";
- wait for confirmation of successful switching;
- wait for the “Empty Road” information from the Video Decisor and the control room;
- switch one of the two integrated actuators to the “Access Allowed” state.

**Figure 7:**
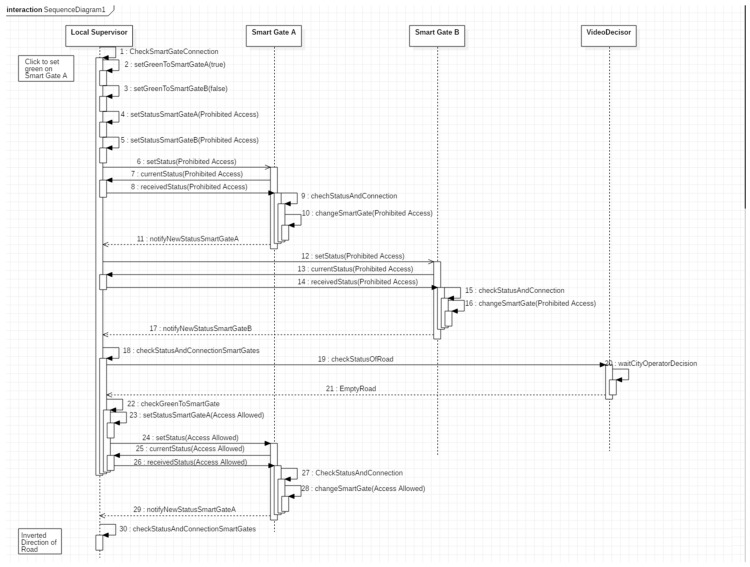
Sequence diagram of operations that are performed in the phase of reversal of the road.

These operations are described in detail in the sequence diagram in Figure 7. 

The first operation that starts the data flow is done by the operator who presses one of the two reverse gear buttons (at the top with the arrow shown in Figure 6). The system (1) checks if the connection with the smart gates is active and (2,3) records the target status that the smart gates will have according to the button selected by the operator. 

It (4,5) saves locally the status “Prohibited Access” for both the smart gates and it (6,12) sends the “Prohibited Access” signal to both smart gates and waits to receive from them an ACK message with the status they intend to change: in this case a “Prohibited Access” ACK must return (7,13). Once this ACK has been received, the system (8,14) responds with a second ACK containing the status “Prohibited Access”. 

If any ACK is lost, a message is shown on the screen indicating the possible inconsistency of the smart gates respect to the local supervisor and the smart gates show “Prohibited Access” until they receive another command and the “Smart Gate Change” protocol starts again. 

If all the ACKs arrive at their destination, the Smart Gates (9–10, 15–16) turn to status “Prohibited Access” and they send a notification of the change made (11, 17, 18). The Video Decisor waits for the video to notify that the road is empty by the control room (19,20). When the “Empty Road” signal is notified (21), the local supervisor (22) reads the target status that the Smart Gates should have, in accordance with the operator’s choice, and the “Allowed Access” signal is sent to the correct smart gate. 

The same “Smart Gate Change” protocol as described above is started with this target Smart Gate (23–30). If the ACKs reach their destination, the local supervisor notifies that the reversal has been successful. If any ACK is lost, a message is displayed on the screen indicating the possible inconsistency of the smart gate respect to the local supervisor and the smart gate show “Prohibited Access” until they receive another command and the “Smart Gate Change” protocol starts again. 

In addition, to increase the security of the reversal protocol, every two seconds the smart gate send their status to the local supervisor and if this does not arrive, the city operator is informed that the smart gate that did not send the status is currently, and it is not online anymore, to the local supervisor. 

To avoid that the status is temporally misaligned with what was present at that time on the smart gate a timestamp is also added to the message. If the timestamp has a misalignment of more than 5 s, then the Smart Gate status may be inconsistent with what is present on the local supervisor and a message is shown to the city operator. For the real-time requirements described above, this time is acceptable for the correct operation of the system. In case it is higher the system is still in a security situation for how the system was developed (smart gates remain in their initial state or both remain on “Prohibited Access”), but a message is shown to the operator to notify him that something needs to be fixed.

**Figure 8:**
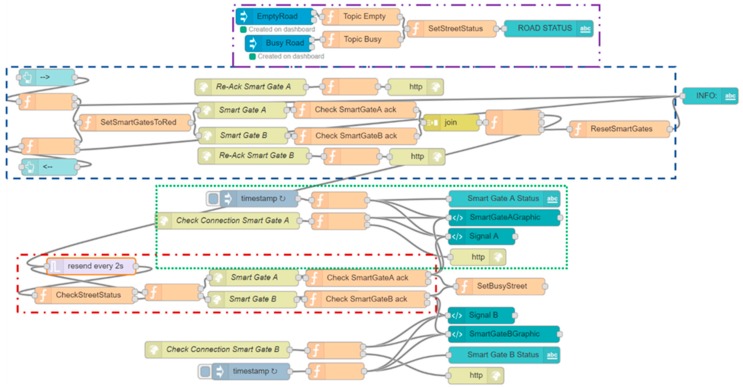
Complete flow of Node-RED that allows to realize the “Smart Gate Change” protocol.

**Figure 9:**
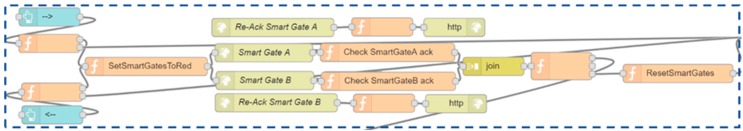
First sub-flow that allows the city operator to set on both smart gate a "Prohibited Access" message. The entire flow is shown in Figure 8.

**Figure 10:**
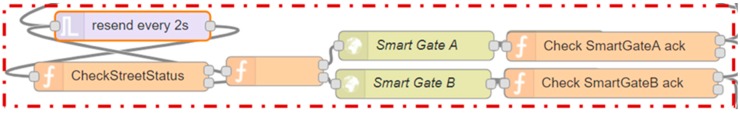
Second sub-flow that controls whether the road is marked as free and sends the "Access Allowed" message to the correct Smart Gate. The entire flow is shown in Figure 5.

**Figure 11:**
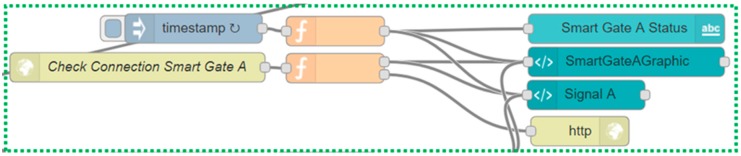
Third sub-flow that controls the consistency of status between the smart gates and the local supervisor. The entire flow is shown in Figure 8.

The protocol described above has been implemented in the smart gates and in the local Supervisor through the Node-Red platform via a Node-Red flow (see Figure 8) which can be divided and explained in sub-flows (see Figure 9, Figure 10 and Figure 11) for greater clarity.

The first sub-flow segment (see Figure 9) allows the local supervisor to inhibit access from both sides of the road before allowing it on the chosen smart gate.

The flow starts with the two blue nodes on the left that correspond to the buttons (present in the interface of the local supervisor) that allow the city operator to reverse the direction of travel. 

Depending on which of the two buttons is pressed, the first operation carried out by the following two orange nodes is to check that both smart gates have sent their signal within 5 seconds from when the button was pressed (1). If this is not the case, a message will be displayed indicating “Connection Problems” to the smart gates. If there are no problems the information, about which of the two smart gates will allow access after being inhibited by both smart gates, is saved (2,3). The following node sends the “Prohibited Access” signal to both smart gates, waiting in response for the ACKs containing the status that was sent (4–17). The last block (18) checks that both ACKs have returned, otherwise it signals connection problems between the local supervisor and the smart gates. If the ACKs are returned but not correct, an inconsistency error is reported, and the smart gates remain in the “Prohibited Access” state waiting for a check or for a new signal to be sent that can be successful.

If the ACKs have been returned and are both correct then the flow continues, going to check if the state of the road is free (19). The check is carried out every 2 seconds (see Figure 10 nodes named “resend every 2s” and “CheckStreeetStatus”) and if it is successful and the road is empty (20,21), the status, that indicates which of the two smart gates should allow access, is recovered (22). The 2 s period has been chosen so as not to overload the system of requests for control of the free road, as if the state of free access is entered two seconds after the occurrence of the event free road should not bring a significant discomfort to drivers. A signal is sent to the right smart gate with the status of “Access Allowed” and is checked again that the ACK returns and that it is correct, if this happens the message that the reversal is successful is shown (23–30). If this is not the case, a message notifying the likely inconsistency between the local supervisor and the smart gates is displayed. This inconsistency should be cleared by periodically sending the statuses running the smart gates to the local supervisor every two seconds. 

It is possible to notice the nodes that have a “Re-Ack” at the beginning of their name, through these nodes it is possible to receive the ACK and to resend the known status and the timestamp of the local supervisor to the smart gate (receivedStatus of the Sequence Diagram shown in Figure 7) that will answer with the last ACK (the one checked in the nodes whose name starts with “Check SmartGate”, notifyNewStatusSmartGateA in the Sequence Diagram shown in Figure 7).

**Figure 12:**
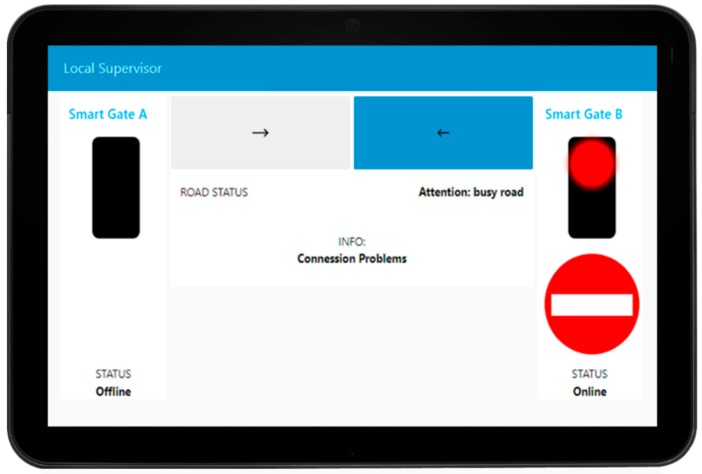
The local supervisor interface in case of inconsistency and following a connection problem with the smart gate A.

The third flow segment (see Figure 11) implements the part that maintains the consistency between local supervisor and smart gates (here is shown just a synthesis of the flow for smart gate A, but the flow for smart gate B is identical). Every two seconds the smart gate A notifies its status to the local supervisor through a direct call that arrives on the node “Check Connection Smart Gate A”. The data that arrives is the status of the smart gate and the timestamp of when the message was created: the status is sent to the dashboard to maintain or recreate consistency and the timestamp is saved. Periodically, the lower part of the flow checks that the timestamp sent by the traffic light and the one saved is no older than a predefined time. If this time is exceeded, the local supervisor interface (see Figure 12) will notify the city operator that a connection with the smart gates no longer exists and it will remove the signals related to the latter because we cannot know if they are any more consistent.

**Figure 13:**
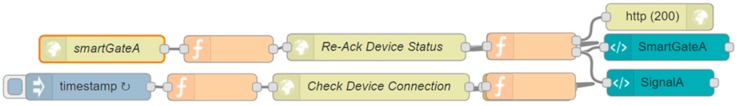
Flow that realizes the logic of the smart gate. The upper part is the one that receives the status and changes it if the ACK exchange is successful. The lower part is the one that checks if the connection is always active between the local supervisor and the smart gate.

The flow segment (see Figure 13) is used on the smart gates for the logic complementary to the one written above is as follows. From the first node at the top the calls are made by the local supervisor when the status of the smart gate must be changed. When a status change message arrives, the new status is saved and is sent to the local supervisor to make sure that the message has arrived correctly. If the return ACK contains the same new status as the one already sent in the first message, then the smart gate changes its status. The bottom flow checks every two seconds if the connection with the local supervisor is active. If not, the status is set to “Prohibited Access” until a city operator intervention or resolution of the connection problem. 

**Figure 14:**
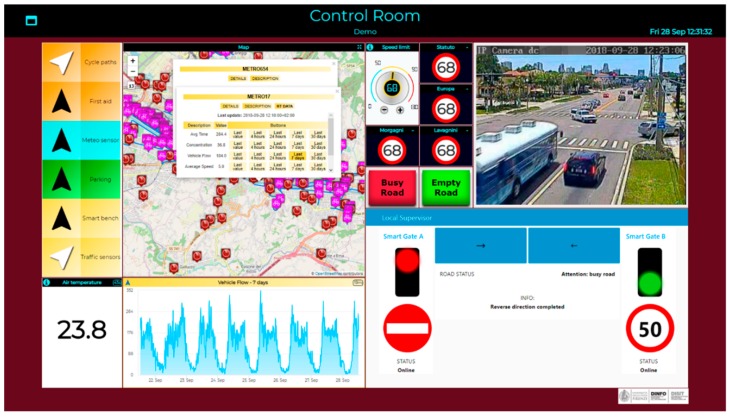
The control room shows a large amount of information and actions that the city operator can do through this dashboard. It allows to have static and real-time information (including historical data) of the POIs that are in the part of the map visible on the left. It allows to send data to the logic developed via Node-red through direct calls (local supervisor view), through web sockets (buttons "Empty Road" and "Busy Road") or through IoT brokers (dimmers and road signs).

The system described above allows to control the travel’s direction of the road being in front of the local supervisor and does not allow any remote control. Using the tools developed and made available by the Sii-Mobility architecture, it is possible to create a simple and intuitive dashboard as shown in Figure 14. Through this dashboard it is possible to remotely control the reversal of the considered road, having much more information at our disposal than if in front of the local supervisor. It is possible to have the real-time images available from the Video Decisor to make sure that the road is free during the reversal, it is possible to modify the signals shown in the smart gates and it is possible to interact directly with the local supervisor to launch the command that starts the reversal protocol described above.

**Figure 15:**
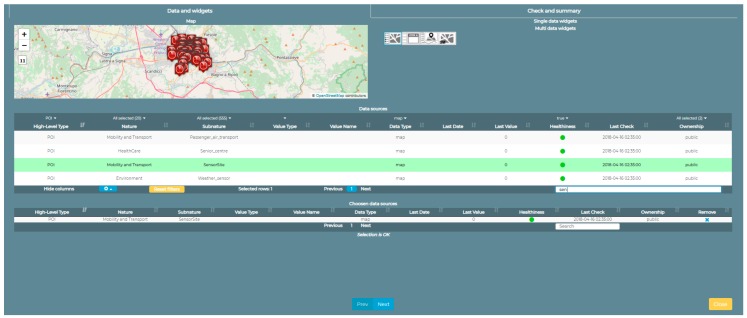
Creation of a widget (the map with the selectors of the various POIs) through the Wizard. In the example, traffic sensors are selected as POIs.

On the left of the image in Figure 14 we can see how it is possible to retrieve information from all the static and real-time data saved by the Sii-Mobility ecosystem. A map can be inserted with points of interest that can be added or removed dynamically by pressing the buttons on the left of the map. For example, in the case of the study presented here, the operator in the control room can show the position and the real-time values of the traffic sensors in the entire area affected by the change in gear of the road with the system installed. Moreover, for each single sensor it is possible to obtain, in addition to the real-time value of the sensor, also the historical data up to one month before the request. Once the choice has been selected, the trend of that sensor in the selected period is shown in the bottom panel. The use of this Widget is done through a Dashboard Wizard (see Figure 15), that utilizes the services provided by a dashboard builder module and allows the user who is creating the dashboard to decide which are the services he wants to see inside the map and on where the area should be centered.

In the top right corner of Figure 14 it shows the signal received by the camera part of the Video Decisor subsystem and based on which it is possible for the operator to decide if the road is free or not. Next to the video there are two buttons that allow the operator to send the signal to the local supervisor whose interface is shown in the lower part of the control rooms. The connection with the interface is sent to an Android device located in the box on the side of the road segment and the operator in the control room can act as if he was in front of the local supervisor. In this way, if the connection is lost, it is not possible to send signals to the smart gates as they cannot access the interface.

**Figure 16:**
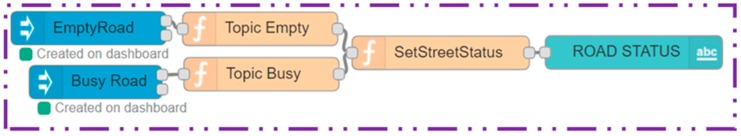
Forth sub-flow that allows the interaction of the dashboard with Node-red. Through this flow, data can be sent from the buttons in the control room to the logic that resides in the local supervisor. The entire flow is shown in Figure 5.

The “Empty Road” and “Busy Road” buttons are part of the Video Decisor subsystem. The importance of these buttons is to make a dashboard in the control room interactive: it allows the city operator to send commands that can be read and, basing on the value that these commands have sent, to execute the linked logic. These nodes were added to the dashboard directly by creating the flow on node-red. In fact, using the blue nodes that can be seen on the left of Figure 16, the user can decide in which of his dashboards add buttons that, if pressed, will send a message in the flow of node-red outgoing from the respective blue nodes. When the flow is executed the button appears on the selected dashboard and sends data to the node inside the flow.

In the development of the subsystem of the Video Decisor the flow, that follows the reception of data from the buttons, controls which of the two buttons has been pressed and saves the value of free/busy road. It will be used to allow the change of state of a chosen smart gate.

**Figure 17:**
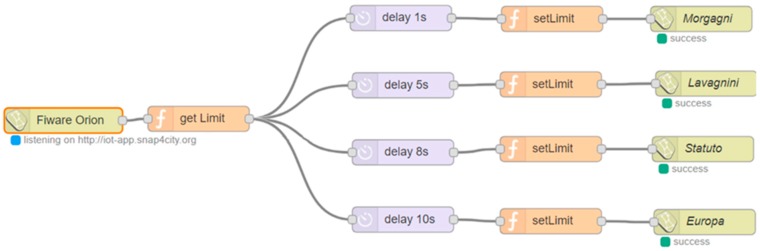
Flow that allows the city operator to read from an entity connected to the dimmer and write these values to four other entities connected to the road signs in the control room. These entities reside within an IoT broker.

Moreover, in addition to the impulsive buttons, other actuators can be inserted in the dashboard for example a dimmer: an example is shown in Figure 14 that present a button that is between the 4 road signs and the map. This dimmer is connected to an entity on a context broker and when it is turned around by the users, the value of the connected entity changes. The detailed flow in Figure 17 allows to read the value of the entity associated with the dimmer in event-driven mode and write the four entities associated with road signs in the dashboard that will change their value based on that set by the dimmer.

## 5. Conclusions

The way the user moves around the city dramatically changed in the last twenty years and the increase of integration between the city infrastructure with the sensors/actuators that are able to act-on/catch its status is enabling nowadays a wide range of new application to be built on top of new IoT architectures and platforms. A set of requirements, these architectures have to be satisfied in terms of smart mobility, has been identified and a brief description of the logical separation between local computation and cloud elaboration has been presented. The major building block of our IoT solution has been explained and a brief description of the main functionalities of the tools have been highlighted.

The development of the experiment discussed above, about a dynamic switching system of a one-way variable road segment, was realized in the context of the Sii-mobility research project to evaluate and demonstrate the feasibility and the flexibility of the IoT platform realized internally in our laboratory. The experiment was proposed directly from the city hall’s operators that are part of the project to check if the platform is mature enough to be use in a real use case scenario of their needs. The architecture of the platform as discussed in the second chapter is modular and the setup of the build blocks was defined step-by-step.

We decide to use IoT Devices based on Raspberry PI since it was the best solution for rapid prototyping and for quick debugging/development of the complete scenario, which required complex sequence of messages exchanged to support high safety level due to the chosen context. After prototyping phase, some hardware components of the setup could be substitute with more compact IoT Devices such as the ones based on the Arduino platform, easy interchangeable since all of them enable the same secure communication towards the other components of the platform and can support a similar wide gamma of sensors and actuators. For the video surveillance sensoring part, a Raspberry IoT Device could be substitute with ad-hoc board with video camera embedded on top, easily available on the market, just taking care to encapsulate the video stream in a protected secured channel conforming our IoT architecture specification.

The complete scenario focus on the communication between the IoT Devices, the control room, and the dashboards and was simulated in a test-lab, where a set of final users was able to test the functionalities and usability of the dashboards that simulate the road messages’ boards. Behind the scene, a city operator was able to dynamically manage the road segment on its purpose, to demonstrate the communication schema and all the possible scenario. All the communication between the IoT components has been kept private using a secure transmission channel using HTTP over TLS/SSL and mutual authentication between the IoT device and the IoT platform. It was successfully possible to integrate the certificate managements (for HTTPS mutual authentication) directly inside all the supported IoT device (esp8266 microcontroller, Arduino and Raspberry), providing the highest possible fidelity level on security end-to-end. The only attack that an intruder can make is acting directly on the hardware with physical/manual hacking, issue that was out of the scope of the current research but that will be target for future works. The final users and the city operators acceded the IoT platform using personal credentials generated upon proper user registration with the platform tools based on the OpenID Connect standard. A common single sign-based on the Keycloak suite was successfully used to enable a common entry point to user authentication. In about six months of testing we has about around 150 final users registered on the platform and in the next 9 months, in the process of the third phase of the Select4Cities challenge (an European project where the developed IoT platform will be part of the trial), around one thousand real users will be involved in a more important stress test of the platform and its main functionalities. On the other side, a test on field of the switchable road segment system is strongly needed to validate the thresholds defined for the IoT application and the impact of lags and related delays of transmission, and to assure all of the main functionalities will not be compromise and the local IoT solution fallback will still enable an operator to act directly on the road with or without the support of the control room.

Even if several manufactories provide ad-hoc sensor system for mobility that can be integrated in the city infrastructure, none of them support a flexible IoT software platform, where the components can be programmed dynamically on the way, but all of them require very specific software able to support an extremely small number of scenarios. For that reason, a complete comparation with the sensor’s manufactories is difficult. A comparison between the available IoT platform, on the other way was possible, trying to analyze their main functionalities (Open Source end-to-end, IoT scalability and execution, visual programming, multidomain semantics, integrated community management, security end-to-end, H24/7 dashboards, multi-protocol on IoT) compared with the one proposed in this research (see Table 1).

Future work would be in extending the usage of the solution on different domain for example in the areas of domotics and Industry 4.0. In those scenarios, the usability of the solution for non- expert users would be stressed

## Figures and Tables

**Table 1:** Comparation of our IoT platform with the more prominent one (bracket symbols mean partially supported).

	Opensource	Scalability	Visual Programming	Multi Domain Semantic	Integrated Community	Security end-to-end	Dashboards	Multi Protocol
Sii-mobility	Y	Y	Y	Y	Y	Y	Y	Y
AWS	N	Y	N	N	N	Y	Y	Limited
Azure IoT	N	Y	(Y)	N		Y	Y	Limited
Fiware	Y	Y	N	N	N	N	Y	Y
IBM	(N)	Y	Y	Y	N	Y	Y	Y
Google IoT	N	Y	N	N	N	Y	N	Mqtt Http
